# Response to Accidental Versus Abusive Head Trauma in Infancy: Is Revival Shaking the Missing Link?

**DOI:** 10.1002/ccr3.9609

**Published:** 2024-11-24

**Authors:** Chris Brook, Waney Squier, Julie Mack

**Affiliations:** ^1^ Faculty of Science Universidad de La Laguna La Laguna Spain; ^2^ Previously Department of Neuropathology John Radcliffe Hospital Oxford UK; ^3^ Radiology Penn State Hershey Hershey Pennsylvania USA

Dear Editor,

We appreciate the opportunity to respond to the concerns raised regarding our recent case report *26 cm fall caught on video causing subdural hemorrhages and extensive retinal hemorrhages in an 8‐month‐old infant* [1].

We agree with the author of the Letter that aging of retinal hemorrhages is not precise, which is why we did not use language that portrayed certainty regarding this issue. Instead, we stated that the evidence “indicates” the RHs occurred “around the time” of the fall and it is “reasonable to attribute” them to the incident. We did not mean to imply that the findings regarding the RHs can definitively attribute the RHs to the fall. However, we point out that there is no evidence of any other event prior to the infant becoming symptomatic that would plausibly explain the findings of retinal hemorrhage.

The author of the Letter then asserts that “acuteness of RH onset would have been better supported by disappearance of most of them after 1 week, rather than the observed persistence.” We are not sure why the author makes this assertion. The longer the RH persisted, the more likely it is that they were acute at the time they were first found.

The issue of whether the retinal folds are “typical of acute traumatic retinoschisis” is also raised. Is the author suggesting that there is a non‐traumatic cause of the retinal fold in this case? We do not believe there is sufficient evidence to accurately determine cause by reference to the “type” of retinal fold, and found no such evidence in the articles cited.

The Letter then raises concerns about the period immediately following the recorded incident, noting that “the video ends abruptly”, and raises the possibility of revival shaking. We clarify that after the fall, a worker at the creche picked up and comforted the infant, and this was captured on video. The baby was not subjected to revival shaking. We also clarify that the mother arrived between 15 and 30 min after the fall, at which time the infant was lethargic and lacked focus in the eyes, presumably signs of concussion.

The Letter suggests that video evidence should follow the infant from the time of the accident to the time of passing the infant to medical care. We wonder if the author applies the same evidentiary requirements for establishing that shaking can cause the findings commonly associated with abusive head trauma. We are not aware of any videotaped shaking event that has resulted in such findings (either violent or in revival attempts). Nor are we are of any independently witnessed shaking event that has led to such clinical findings in a healthy infant. If the evidentiary requirement for these cases was an extended videotape until delivered to medical care, then no case or case series would have ever been published in the field.

The author of the Letter also discusses the historical narratives of shaking done by caregivers in order to revive or resuscitate the infant. We agree that such narratives are common, and are often dismissed or wrongfully interpreted as being confessions. However, with respect to shaking in revival attempts, we do not know how cerebral or ocular findings could be attributed to the act of revival shaking rather than to whatever caused the collapse in the first place.

Finally, the author of the Letter questions whether our study helps clarify the pathogenesis of ocular or cerebral hemorrhage. Our case study should not be read in isolation, but as adding to the growing list of cases compiled over the past decades that, taken as a whole, provide strong evidence that short falls can result in both cerebral and extensive ocular hemorrhages.

## Author Contributions

**Chris Brook:** writing – original draft. **Waney Squier:** writing – review and editing. **Julie Mack:** writing – review and editing.

## Data Availability

The authors have nothing to report.
